# Recommendations to Deliver Person-Centered Long-Term Care for Persons Living With Dementia

**DOI:** 10.1016/j.jamda.2021.05.003

**Published:** 2021-05-25

**Authors:** Laura M. Wagner, Kimberly Van Haitsma, Ann Kolanowski, Joanne Spetz

**Affiliations:** a Philip R. Lee Institute for Health Policy Studies and the UCSF Health Workforce Research Center on Long-Term Care, University of California, San Francisco, San Francisco, CA, USA; b College of Nursing, The Pennsylvania State University, State College, PA, USA

**Keywords:** Person-centered care, outcome assessment, dementia care workforce, long-term care

## Abstract

Person-centered care (PCC) is the standard for the delivery of long-term services and supports (LTSS). In this article, we summarize the state of the science on meaningful outcomes and workforce development and discuss what is needed to ensure that person-centered LTSS becomes a universal reality. These 2 themes are intimately related: the dementia care workforce’s capacity cannot be improved until care processes and outcomes that are significant to PCC are explicated. The LTSS workforce needs training in PCC as well as pragmatic measures to assess the quality of the care they provide. We conclude with several recommendations for future policy and practice-oriented workforce research.

There are more than 5 million people living with neurodegenerative diseases in the United States, conditions that have no cure and require ongoing services and supports to sustain a level of safety and function.^1^ Person-centered care (PCC) is the standard for the delivery of long-term services and supports (LTSS) to people living with dementia.^2^ PCC is characterized by shared decision-making between the individual and relevant providers where the person’s values and preferences guide aspects of their health care and support the person’s health and life goals.^3^ LTSS encompass a range of medical and personal care assistance when illness or disability interferes with self-care. LTSS is complex, provided by myriad agencies, organizations, and individuals, which often function independently of each other. The workforce delivering services is large, represents many different levels of training and formal education, and varies in its composition and adequacy across organizations and regions. Organization context, workforce and staffing, approaches to PCC, and outcomes are important domains to consider when developing LTSS programs to support thriving environments for people living with dementia.^4–6^

The variability found within LTSS is matched by the growing racial and ethnic diversity of the US population, including people living with dementia. What has not kept up with this burgeoning and complex system is research that clearly identifies outcomes of greatest importance to the diverse groups of people living with dementia and their care partners, the LTSS processes and approaches that achieve their individual goals with a focus on remaining strengths rather than deficits, and the type of workforce that is needed to deliver these services.

In this article, we summarize meaningful outcomes and workforce development for dementia care and discuss what is needed to ensure that person-centered LTSS becomes a universal reality. These themes of outcomes measurement and the workforce are intimately related: the dementia care workforce’s capacity cannot be improved until care outcomes and processes that are significant to impacting person-centered care are explicated. The LTSS workforce needs training in PCC as well as pragmatic measures to assess the quality of the care they provide. We conclude with several recommendations for moving the state of the science forward.

## Assessing the Delivery of Person-Centered Care

A recently released report from the National Academies of Science, Engineering and Medicine reviewed more than 5 decades of the state of the science regarding promising evidence-based approaches to support people living with dementia, most of whom live in the community and may benefit from LTSS.^7^ In this review, the authors noted that a host of nonpharmacologic interventions have demonstrated some encouraging impacts on clinical outcomes. For the person living with dementia across all community-based and institutionalized LTSS settings, these approaches seek to address symptoms primarily in 3 domains: cognitive decline, functional decline, and behavioral expressions of distress. Some of the approaches for which evidence is emerging include cognitive stimulation training (eg, reality orientation), exercise, in-home modification, family caregiver skills training, music and animal therapy, and massage and touch.^7^ Overall, this large body of research has demonstrated the potential for a wide variety of nonpharmacologic interventions to effectively treat, manage, and even prevent some of the negative outcomes related to living with dementia.

There also is an even larger body of work focusing on outcomes related to care partners/caregivers.^8^ To date, we know that there is demonstrated efficacy for more than 200 multicomponent family care partner/caregiver supportive approaches and programs geared toward LTSS.^9^ These approaches have demonstrated at least a mild to moderate effect on care partner outcomes such as well-being, communication, decreased burden, and distress.^7,10^ Some examples of these approaches include psychoeducation, social support, and respite. Toolkits and resources that include these elements are becoming more widely available through Web sites and supportive organizations, for both family care partners (such as https://bpc.caregiver.org/) and for staff care partners providing care in long-term residential environments (such as https://nursinghometoolkit.com).

Despite this encouraging body of work, we still have much to do. First, in spite of the promise of evidence for these care approaches for people living with dementia, recent reviews have suggested a number of ways in which we can still improve our scientific rigor.^7,8,10^ One area for improvement involves adapting interventions for application in ethnically and racially diverse populations.^11^ Second, we need to consider the long-term effects of interventions and the relationships between near-term endpoints and long-term outcomes. This demands a better understanding of the effective mechanisms of action for each intervention, which is critical to advancing intervention science to the care approaches related to persons living with dementia. We lack an understanding of how to make many of these evidence-based interventions pragmatic enough for implementation and dissemination into the real world of care.^12^ The National Institute on Aging IMPACT Collaboratory^13^ (https://impactcollaboratory.org/) has taken this issue head on. Finally, there is a need to expand our focus to a broader range of outcomes that matter to people living with dementia and their care partners.^14^ Evidence-based care approaches have focused on outcomes identified by researchers, which are frailty- and deficit-focused,^4^ rather than on how to best optimize the experience of living with dementia, considering what most people want as their goal in life, which is to flourish. Given the enormous amount of work that creates a clearer picture of optimal dementia care, we are now at an ideal point to consider more carefully what outcomes we should be seeking to affect.

When we consider what outcomes matter to a person living with dementia and their care partners, we should center on what constitutes a successful impact of an evidence-based approach and what elements of care delivery are essential to produce that impact.^15^ Honoring what matters most to the individual living with dementia is critical in guiding the development of person-centered approaches and how we measure the impact of interventions.

To accomplish this goal of considering a broader range of outcomes that matter to people living with dementia, we need good models or frameworks to guide and lend rigor to our thinking.^16^ Many models exist, but for illustrative purposes, we will consider the utility of the Good Life Model originally developed by Lawton and recently adapted by Gitlin and Hodgson.^17^ This model guides thinking around assessment of care delivery and outcomes of optimal dementia care. It maintains that to give the best support to people living with dementia we must consider at least 4 interrelated quadrants of life: psychological well-being, behavioral competence, perceived valuation of life, and the objective environment. This model allows us to systematically identify the categories and range of outcomes that may be relevant to the lived experience of people living with dementia, including positive affect, hope, joy, having a sense of purpose, personal growth, and dignity.^18–21^

The Dementia Care Practice Recommendations framework delineates 9 types of care processes.^22^ These processes include the following: PCC; detection and diagnosis; assessment and care planning; medical management; information, education, and support; ongoing care for behavioral and psychological symptoms of dementia, and support for activities of daily living; staffing; supportive and therapeutic environments; transitions and coordination of services; and are considered to be important by people living with dementia and their care partners^22^ and which can support person-centered outcomes. Models and frameworks such as these are critical to focusing future research efforts to identify areas for new measurement development, guide our thinking regarding mechanisms of action, and ultimately develop evidence-based approaches that impact outcomes that matter most to people living with dementia.

Some research is considering measures that fit the criteria of capturing impactful intervention outcomes as well as essential processes of care that have been identified as meaningful outcomes from the perspective of the person living with dementia.^19^ Some of these measures are self-reported, and a recent review of emerging positive psychology outcome measures by Stoner and colleagues^23^ included a variety of nascent outcome measures based on positive constructs such as identity and hope. In addition, research is emerging that focuses on the utility of observational measures such as the construct of affect balance.^24–26^ Originally developed by Bradburn^27^ this construct suggests that our focus be on the balance or ratio between positive and negative experiences of the person receiving care. This is particularly attractive in the context of dementia, where completely eliminating negative aspects of living with the disease may not be possible. There also has been advancement in the measurement of care processes, including measures such as preference congruence.^28,29^ Preference congruence is a pragmatic process measure that assesses how well care processes are aligned with the everyday preferences expressed by the person living with dementia. This measure has shown some promising feasibility within long-term care communities to facilitate the delivery of care that is truly person-centered.

## Advancing the LTSS Workforce

The care partners of those living with dementia include both unpaid and paid individuals. The paid workforce is large and diverse, employing millions of people,^30^ most of whom are in occupations that do not require a college degree. The largest occupation by far is the “direct care” workforce, which consists of 2.4 million personal care aides, 1.1 million nursing assistants, and 800,000 home health aides. Direct care workers provide services across care settings including in skilled nursing facilities and nursing homes, residential care communities and assisted living, home health, household employment, and personal care services agencies. Job growth is expected to be very high over the next decade for direct care workers, estimated by the US Bureau of Labor Statistics at 36% for personal care aides and more than 36% for home health aides.

Although most of the jobs in LTSS do not require more than a few weeks of training, there may be benefits to expanded training for direct care workers, particularly pertaining to providing PCC to people living with dementia. Many questions remain, including whether training affects the care team’s effectiveness, how to best integrate care partners with primary care geriatricians and other specialists, and whether training leads to cost savings.

Some preliminary research suggests that worker training can produce better outcomes for people living with dementia, greater skills and confidence among care partners, and lower costs. The Centers for Medicare and Medicaid Services (CMS) Innovation Center supported projects in California, Arkansas, and South Carolina that aimed to train personal care workers^31^ in specific skills that varied across projects.^31,32^ Workers were very satisfied with the training they received, reported that they gained caregiving skills and knowledge, and felt more confident in their work. Those receiving care perceived quality of care was higher after workers received training; unfortunately, the evaluations were limited in the person-centered outcomes measured. In California, the training was associated with decreases in emergency department visits and hospitalizations, which produced cost savings^33,34^ but no cost savings were gained in South Carolina. Each of the pilot projects was small; further research is needed to assess how different training approaches could be scaled to larger numbers of workers and what the full range of impacts such training has to improve PCC for people living with dementia and their care partners.

There is also a lack of evidence regarding whether and what types of training for direct care workers are associated with improvements in person-centered or worker outcomes. Federal requirements differ by job classification and employment setting; for example, federal regulations require that nursing assistants who work in nursing homes and home health aides who work in home health agencies (providing shorter-term post-acute care) have 75 hours of training, but there are no federal training requirements for personal care aides. Seventeen states require that home health aides complete more than 75 hours of training,^35^ and 30 states require that nursing assistants complete more than 75 hours of training.^36^ For personal care aides working in Medicaid-funded LTSS programs, 11 states had no training requirements at all in 2014 and 21 states had different training requirements for different specific programs within Medicaid.^37^ There has been no research on whether these differences in training requirements are associated with differences in outcomes for people living with dementia or people receiving LTSS in general. National guidelines, such as core competencies for home health aides developed by the Centers for Medicare and Medicaid Services, also have not been evaluated. The lack of evidence and differing training requirements within and across states creates a confusing situation for workers, agencies, and policy makers.

Another topic about which we know little is how regulations that delineate which services personal care aides are allowed to perform affect people living with dementia or people receiving LTSS in general.^38–40^ Some states have very restrictive regulations, not allowing direct care workers to administer any medications, help with insulin injections, or engage in most care tasks such as these. In contrast, other states permit direct care workers to provide a wide range of supportive services and, in some states, to have the person they support fully direct the services they provide.^41^ This latter approach, called a consumer-directed program, is a component of the Medicaid programs of 36 states and is well-aligned with the goals of PCC,^42^ but there is variation across states in the populations eligible for consumer-directed care and the provisions under which these programs operate. States that restrict the practice of direct care workers do so because they believe that such restrictions are necessary to protect clients, but there is little research on whether such regulations improve outcomes or safety. Recent qualitative research reported that people receiving support from personal care aides may be more likely to be forced to move into facilities for care when restrictive laws limit the ability of their aides to meet their care needs.^43^ More research is needed to understand the impact of these regulations and which regulations are most beneficial to providing PCC for people living with dementia and their care partners.

Although direct care workers are the largest occupations providing LTSS, interprofessional teams are essential to PCC for people living with dementia and their care partners. Further research is needed about how to enhance the capacity existing personnel and integrate them into interprofessional person-centered care teams.^44^ The health care system is experiencing a shortage of geriatricians and these shortages are likely to worsen in the future. New occupations such as dementia care specialists^45^ can play an important role in expanding the support and care available to people living with dementia, as can approaches that integrate direct care workers into the care team. The educational preparation of a dementia care specialist consists of training on basic and general dementia knowledge and skills (Tier 1) followed by advanced-level training to increase knowledge and self-efficacy (Tier 2), and huddle calls to problem solve difficult situations.^45^ Improving communication and coordination among team members and with the person living with dementia is essential for PCC, but there is little evidence that links promising approaches with person-centered outcomes or on how to scale up promising practices.

There may be potential for assistive technology solutions to support improvements in the support provided to people living with dementia and their care partners. Such technologies are proliferating in LTSS, but many technology developers are paying little attention to how their products interact with the workforce or their appropriateness for people living with dementia.^46,47^ Creation of valuable person-centered technology tools requires that product developers work with the target population and their care partners, and it would be ideal if they also rigorously evaluated person-centered outcomes and process measures.

A final area of great importance is supporting the LTSS workforce in its interface with an increasingly diverse population of people living with dementia. Establishing training^48^ and ongoing support to advance principles of cultural humility and PCC is challenging when the workforce includes a heterogeneity of occupations and workplace settings and the population of people living with dementia lives within myriad cultural backgrounds. Some innovative model programs have been implemented, such as Stanford’s iSAGE Ethnogeriatrics training.^49^ This Web-based program aims to provide learners with training in the principles of successful aging, with designed to develop expertise in working with specific ethno-cultural groups, such as African Americans, Asian Indian American, Hispanic/Latino American, and Alaska Native individuals.^50^ Such programs need to be adapted to multiple settings and communities and evaluated to identify best approaches to maximize their benefit for PCC for people living with dementia.

## Recommendations for Practice and Policy Focused Workforce Research

The evidence generated to date has shaped some approaches to PCC for people living with dementia and their care partners, but there are numerous areas for research that should be prioritized (see Table 1), including the following:

1. Develop and implement person-centered outcome measures that are informed by the perspective of people living with dementia, holistic in focus, strength-based in nature, and can be used pragmatically across long-term services and supports settings.
2. Use theory-driven frameworks to develop and test multilevel evidence-based approaches that address the complex heterogeneous and interacting challenges experienced by people living with dementia and their caregivers over the full course of the disease; for example, approaches can be guided by socioecological levels, and heterogeneity can reflect cultural diversity.
3. Enhance intervention research by incorporating components that address social determinants of health (eg, social and economic resources) and examining the extent to which these determinants moderate or mediate intervention outcomes for people living with dementia and caregivers.
4. Assess how interventions’ effect on intermediate endpoints (eg, cognition, function, well-being) relate to longer-term outcomes including caregiving intensity, caregiver health, movement into residential long-term care, and costs to individuals, families, and society.
5. Develop and evaluate training for direct care workers to identify specific competencies and modalities that best contribute to improved health, quality of life, financial, and social outcomes for people living with dementia and their caregivers.
6. Analyze the impact of racial and ethnic diversity among people living with dementia and the health workforce providing their care, and develop and test approaches that promote cultural humility, cultural competence, and communication skills.
7. Determine the relative effectiveness and efficiency of different interprofessional workforce models in providing high-quality care to people living with dementia, and how to support workforce collaboration across home, community, and residential settings.
8. Analyze the interactions between caregivers and health care workers and technologies designed for the care of people living with dementia; determine how technological change will affect future workforce needs, and design and evaluate effective education and training for caregivers and health care workers to use new technologies effectively.
9. Examine the impact of dementia care training and characteristics of the paid dementia care workforce on those health care outcomes that are most significant among people living with dementia. Further research is needed to compare the effectiveness of dementia care training programs. Due to heterogeneity of training, it is not known which components of training are most effective in creating improvements in the quality of care.
10. Research best practices that best support the dementia care workforce, including maintaining adequate staffing levels, staff training, compensation, supportive work environments, career growth and retention, and family engagement.
11. Conduct health policy research to examine the impact of requirements for dementia care worker training, including necessary training hours and instructional content.

## Figures and Tables

**Table 1:** Future Research to Deliver Person-Centered Long-Term Care for Dementia

1. Develop person-centered outcomes
2. Test theory-driven approaches that address care challenges
3. Incorporate social determinants of health into research strategies
4. Assess how interventions impact intermediate- and long-term outcomes
5. Develop and evaluate competency-based dementia care training
6. Analyze the impact of race and ethnic diversity of the workforce,caregivers, and clients
7. Determine effective workforce models of care across professions and settings
8. Analyze interactions between technology and caregivers
9. Examine dementia care training impacts on care outcomes
10. Research best practices that support the dementia care workforce
11. Conduct policy research to examine dementia care training policies
